# Association of health literacy and nutritional literacy with sun exposure in adults using structural equation modelling

**DOI:** 10.1186/s12889-022-13465-6

**Published:** 2022-06-10

**Authors:** Nasim SaeidiFard, Ali Asghar Haeri-Mehrizi, Zahra Akbarzadeh, Nasim Janbozorgi, Ali Montazeri, Mehdi Yaseri, Sakineh Shab-Bidar

**Affiliations:** 1 Department of Community Nutrition, School of Nutritional Sciences and Dietetics, Tehran University of Medical Sciences (TUMS), No 44, Hojjat-dost Alley, Naderi St, Keshavarz Blvd, Tehran, Iran; 2 Health Metrics Research Center, Iranian Institute for Health Sciences Research, ACECR, Tehran, Iran; 3 Faculty of Humanity Sciences, University of Science and Culture, Tehran, Iran; 4 Department of Epidemiology and Biostatistics, Tehran University of Medical Sciences (TUMS), Tehran, Iran

**Keywords:** Health literacy, Nutritional literacy, Sun exposure, Vitamin D knowledge, Attitude toward sun exposure, Structural equation modeling

## Abstract

**Background:**

Inadequate health and nutritional literacy is a common problem among adults, associated with poor health outcomes. Therefore, this study aimed to investigate the relationship between health literacy and nutritional literacy to sun exposure behaviour.

**Methods:**

We conducted a cross-sectional study on 261 adults (18–65 years) in Iran. Data was collected on knowledge, motivation, health literacy, nutritional literacy, and sun exposure behaviour using an interview-assisted questionnaire. Using the information–motivation–behavioural skills model and structural equation modeling, we tested whether health and nutritional literacy were associated with the relationships between knowledge of vitamin D, attitudes toward sun exposure, and sun exposure behaviour. Different models using structural equation modeling were performed to analyze the data.

**Results:**

The finding showed that health literacy (*β* = 0.29, *p* < 0.001) and nutritional literacy (*β* = 0.14, *p* = 0.02) was directly associated with sunlight exposure. Indirect relationships also existed between knowledge and sunlight exposure through health literacy (*β* = 0.33, *p* < 0.001) and nutritional literacy (*β* = 0.22, *p* = 0.01). The model had good fit (x^2^/df = 1.422; RMSEA = 0.040; CFI = 0.851; NFI = 0.657). There was no significant relationship between health literacy and motivation (*β* = 0.11, *p* = 0.16), nutritional literacy and motivation (*β* = 0.06, *p* = 0.42) and motivation and sun exposure (β = 0.01, *p* = 0.91).

**Conclusions:**

The findings showed that individuals with sufficient health literacy and nutritional literacy were more likely to have exposure to sunlight. Health and nutritional literacy should be considered when educating adults about vitamin D supplements and sunlight exposure.

## Background

In recent decades, numerous investigations have reported the prevalence of vitamin D deficiency worldwide [1–3]. Vitamin D deficiency is a widespread health problem in all countries of all ages and both sexes [4]. In Iran, the prevalence of vitamin D deficiency is above 85% [5]. Vitamin D is a fat-soluble vitamin and is an essential nutrient for the body [6], which plays an important role in the health and survival of humans [7]. Several studies have focused on its role in the prevention of diseases such as heart disease [8], inflammatory bowel disease [9], multiple sclerosis [10], rheumatoid arthritis [11], Immune system diseases [12], diabetes [13] and infectious diseases [14]. Vitamin D deficiency worldwide is probably due to limited sun exposure and insufficient intake of vitamin D from the diet [15]. It is well known that higher levels of vitamin D can be achieved through sun exposure [16]. This exposure to sunlight seems to provide enough vitamin D even in the winter, except for those who cannot or do not want to go out of the house. Although many people may have heard about vitamin D, they are unaware of its prominent role and resources. Also, due to cultural and religious factors and prevalent environmental conditions; females are usually at higher risk of reduction in the synthesis of vitamin D [17]. Knowledge about vitamin D and the motivation to sunlight is relatively limited in many societies [18]. Inadequate knowledge and inspiration about sunlight can reduce exposure for sunlight and thus reduce vitamin D intake. In some studies, knowledge, and motivation have been considered influential factors in promoting health behaviours and the development of health and nutritional literacy [19]. Many studies have also suggested that health and nutritional literacy affect knowledge and motivation, which affecting health outcomes [20, 21].

Nutritional literacy is the skill and the ability to access, and understand information about healthy nutrition and use it to have a healthy diet and lifestyle [22]. Previous studies have shown that nutritional literacy is significantly associated with health behaviors among adults [23]. The results of the studies show that increasing knowledge and nutritional literacy has a significant effect on changing people’s diet towards a healthy diet and a suitable nutritional model [24].

Health literacy is a personal and social capacity for access to understanding, assessment, information, and health services, and optimal utilization for the promotion and improvement of health [25]. Based on research, poor health literacy is also associated with more inferior health status [26], poor use of flu vaccination [27], and higher BMI. Inadequate or limited health literacy has now been highlighted as a global problem in many countries.

Health literacy and nutritional literacy may play a role in the relationship between knowledge, motivation, and sun exposure behavior. Since there has not been a study on the relationship between health literacy, nutritional literacy and sunlight exposure, the present cross-sectional study aimed to investigate such relationships using structural equation modeling (SEM) [28].

## Methods

### Study design and participants

This cross-sectional study was carried out on a sample of Iranian adults who referred to health centres in Tehran, Iran from February to July 2018. The inclusion criteria were: being adult men and women aged 18 to 65 years, having reading and writing abilities, and being able to participate in social activities. Participants were selected using two-stage cluster sampling from existing health centres in Tehran. Health centres were divided into five regions: North, South, East, West, and Central. Then a list of health centres in each region was provided, and proportional to the number of health centres in each area, 25 health centres were selected randomly. In all, 300 individuals were approached. Of these, thirty-nine participants dropped out of the study. A total of 8 participants were older than 65 years, 6 were illiterate, and 25 completed the questionnaires incompletely. This sample size for SEM analysis seemed to be enough because the minimum sample size required for such an analysis is 200 [29]. The study was performed in accordance with the Declaration of Helsinki and approved by the ethics committee of Tehran University of Medical Sciences (IR.TUMS.VCR.REC.1396.4028). All participants signed the informed consent. Participants were informed in detail about the study purpose before completing their written informed consent. All survey instruments were read aloud, and responses were recorded by the research fellows. The survey took 90 to 120 min to be completed.

### Questionnaires and measures

1. **Demographic, anthropometric and physical activity**: Demographic questionnaire included information on age, sex, marital status, education level, occupation, smoking status, body mass index, and physical activity. Age was recorded on a continuous scale, and education level was recorded as primary, secondary, and higher. Weight was measured with light clothing and without shoes using a digital scale (Seca 808) and recorded to the nearest 100 g. The height was measured in a standing position without shoes, using the standard Seca stadiometer, recorded with an accuracy of 0.1 cm. BMI was calculated as weight (kg)/height^2^ (m). Level of physical activity was assessed with the International Physical Activity Questionnaire (IPAQ). Data from the IPAQ were used to estimate compliance with guidelines for physical activity presented as low, moderate, and high levels of activity expressed in (MET-h per week) [24].
2. **Health literacy**: We used the Health Literacy Instrument for Adults (HELIA) for data collection [30]. The questionnaire has 33 items with 5-point response categories and measures five dimensions: reading (4 questions), access (6 questions), comprehension (7 questions), assessment (4 questions), and decision making and behavior (12 questions). Scores are classified, and interpreted as 0–50 = inadequate health literacy, and 51–100 = adequate health literacy.
3. **Nutritional literacy**: The Nutrition Literacy Scale (NLS) consisted of 28 items [31]. In general, items within each content area are ordered from the easiest to the more difficult. Scores are classified and interpreted as 0–15 = inadequate nutritional literacy and 16–28 = adequate nutritional literacy.
4. **Attitude toward sunlight exposure**: Sunlight exposure was measured using six items: (1) ‘‘I like sunlight’’; (2) ‘‘I use sunhat when exposed to sunlight”; and (3) ‘‘I use sunscreen products containing SPF ≥ 15 when exposed to sunlight’’ (4) ‘‘I like outdoor activities’’ (5) ‘‘Usually I spend most of my time outdoors’’(6) ‘‘The time I expose myself to sunlight is enough’’ [32].
5. **Knowledge of vitamin D**: Knowledge of vitamin D was measured by five items: (1) ‘‘I have ever heard about vitamin D’’;; (2) ‘‘Vitamin D is good for bone health’’; (3) ‘‘Vitamin D supports calcium absorption’’; (4) ‘‘Vitamin D can be supplemented by sunlight exposure’’; (5) ‘‘The minimum time needed for sunlight exposure is 30 min if we want our body to develop a sufficient amount of vitamin D’’ [32]. Scores were calculated based on a previous study by Boland et al. [33].
6. **Sunlight exposure behaviour**: Sun exposure duration was used to calculate the hours of daily sun exposure over the previous week [34]. There were three choices for the amount of time spent each day outdoors (0 ≤ 5 min, 1 = 5–30 min, and 2 ≥ 30 min) and four choices for clothing or skin exposure while outdoors (1 = face and hands only; 2 = face, hands and arms; 3 = face, hands and legs; and 4 = “face, hands, legs and arms”). A score to estimate of their weekly sun exposure was calculated. The amount of time spent outdoors and the amount of skin exposed was calculated for each day to create a daily sun exposure score. All seven days’ sun exposure scores were summed to obtain the weekly sun exposure score. Scores are classified and interpreted as ≥ 30 representing sufficient sunlight exposure and < 30 representing insufficient sunlight exposure.

### Data analysis

Data were analyzed using R version 3.4.4. A model was developed for the hypothesized relationships among health literacy, nutritional literacy, knowledge of vitamin D, attitudes toward sunlight, and sunlight exposure behavior. In fact, sun exposure behavior was considered as an outcome measure, and the remaining variables in the model were considered as covariates including, knowledge of vitamin D (indicated by five questions), attitudes toward sunlight exposure (indicated by six questions), health literacy, and nutritional literacy. The model’s goodness of fit was determined using four measures of fit: relative chi-square (x^2^/df), normed fit index (NFI), comparative fit index (CFI) and root-mean-squared error associated (RMSEA). Smaller relative chi-square values indicate a better fit, and an insignificant relative chi-square is desirable. Relative chi-square is thought to be less dependent on sample size, and values greater than 1 and below 2 are considered a good fit [35]. NFI and CFI range from 0 to 1, with values closer to 1 representing a very good fit [36]. RMSEA is an index of the degree to which a confirmatory structure approximates the data being modelled and a value less than 0.08 reflects a good model fit [37]. A *P*-value of < 0.05 was considered statistically significant.

## Results

### Participants

In total, a convenient sample of 261 individuals participated in the study. The mean age of participants was 38.8 years (SD = 11.09). The majority of participants were female (*n* = 166; 63.6%), overweight (42.1%), married (73.2%), employed (51.7%), and had a higher education qualification (49.4%). The Participants’ characteristics are shown in Table 1.

**Table 1:** Socio-demographic characteristics of the study sample (*n* = 261)

	No.	Percentage
Age		
Mean (SD)	38.8 (11.09)	**-**
Gender		
Male	95	36.4
Female	166	63.6
Marital status		
Single	64	24.5
Married	191	73.2
Widowed	6	2.3
Educational attainment		
Primary	55	21.1
Secondary	77	29.5
Higher	129	49.4
Occupation		
Employed	135	51.7
Housewife	94	36
Retired	5	1.9
Unemployed	27	10.3
Smoking status		
Smoker	20	7.7
Non-smoker	241	92.3
WHO BMI Category		
Underweight (< 18.5 kg/m^2^)	7	2.68
Normal weight (18.5–24.9 kg/m^2^)	89	34.1
Overweight (25.0–29.9 kg/m^2^)	110	42.1
Obese (> 30.0 kg/m^2^)	21.1	55
Physical activity		
Low	86	33
Medium	108	41.4
High	67	25.7

### Vitamin D knowledge

This study has indicated a high level of vitamin D Knowledge. The majority of participants (83.9%) had heard about vitamin D, 83.1% agreed that vitamin D is good for bone health and 72.4% knew that vitamin D is necessary for supporting calcium absorption, and 69% knew that vitamin D could be supplemented by sunlight exposure. Moreover, 64% of them had information on the minimum time needed to spend outdoors to get enough Vitamin D (Table 2).

**Table 2:** Descriptive statistics of outcome variables

	No.	%
**Knowledge about vitamin D (correct response)**		
Have ever heard of vitamin D	219	83.9
Vitamin D is for maintaining bone health	217	83.1
Vitamin D is for supporting calcium absorption	189	72.4
Vitamin D can be increased by sunlight exposure	180	69
Minimum time for sunlight exposure is 30 min	120	64
**Attitudes toward sunlight exposure**		
Like sunlight	144	55.2
Use sun hat when exposed to sunlight	211	80
Use sunscreen products containing SPF ≥ 15 when exposed to sunlight	168	64.4
Like outdoor activities	177	67.8
Spend most of the time outdoors	55	21.1
Feel that have had sufficient sunlight exposure	83	31.8
**Behavior for sufficient sunlight exposure**	178	68.2
**Health literacy**		
Adequate	212	81.2
Inadequate	49	18.8
**Nutritional literacy**		
Adequate	99	37.9
Inadequate	162	62.1

### Attitude toward sunlight exposure

The level of attitude toward sunlight exposure was relatively low. More than half of the participants (55.2%) responded that they like sunlight, 64.4% indicated that they used sunscreen products with a sun protection factor (SPF) ≥ 15. Moreover, 80.0% said they used a parasol to shade themselves from the sun; 67.8% reported that they like outdoor activities, and 21.1% reported that they spend most of the time outdoors. Overall, 31.8% of the participants felt that they had sufficient sunlight exposure (Table 2).

### Sun exposure behaviour, health literacy and nutritional literacy

The findings indicated that 68.2% of participants spent 30 and more than 30 min outdoors last weekend. Also, we found that the majority (81.2%, *n* = 212) had an adequate level of health literacy (Table 2). Considering the cut-off point for nutritional literacy, 37.9% of the participants had an adequate level of nutritional literacy.

### Relationships between knowledge, attitude, and behaviour (first model)

The first model describes the relationship between knowledge, attitude, and behavior in a direct path. The model fit values (CMIN/df = 1.719, NFI = 0.561, CFI = 0.734 and RMSEA = 0.053), suggest the model has an acceptable predictive ability or fit. The relationship between knowledge and sunlight exposure behavior was insignificant (*β *= 0.05, *P* = 0.555). In addition, attitude toward sun exposure was not directly associated with sunlight exposure behaviour (*β* = 0.05, *p* = 0.472) (Table 3).

**Table 3:** Total effects between variables in the model

	Variables		S.E^a^	P**
Motivation	→	Health literacy	0.34	0.16
Information	→	Health literacy	0.17	0.001
Motivation	→	Nutritional literacy	0.38	0.42
Information	→	Nutritional literacy	0.19	0.01
Health literacy	→	Sun exposure	0.07	< 0.001
Nutritional literacy	→	Sun exposure	0.34	0.02
Motivation	→	Sun exposure	0.19	0.91
Information	→	Sun exposure	0.05	0.47
Motivation	→	Use sun hat	0.95	0.01
Motivation	→	Use sunscreen	0.15	0.03
Motivation	→	Enjoy outdoor activities	0.36	0.50
Motivation	→	Spending time outdoor	0.43	0.06
Motivation	→	Feel have sufficient sun exposure	0.36	0.56
Information	→	Bone health	0.19	0.01
Information	→	Calcium absorption	0.19	< 0.001
Information	→	From sunlight	0.22	< 0.001
Information	→	Time for exposure	0.18	0.10

^a^ *S.E *Standard error

** *P*-value of < 0.05 was considered statistically significant

### Relationships between knowledge, attitude, and behavior through health literacy (second model)

The second model indicates the relationship between knowledge, attitude, and behavior, and the effect of health literacy on this relationship. The model fit indices (CMIN/df = 0.953, NFI = 0.777, CFI = 1.000 and RMSEA = 0.00) indicated a relatively satisfactory model fit to the data. The paths between health literacy and sunlight exposure (*β* = 0.29, *p* < 0.001) and knowledge and health literacy (*β* = 0.34, *p* < 0.001) were statistically significant. Thus, health literacy had a direct effect on sunlight exposure. In addition, results confirmed the expected indirect effect of knowledge on sunlight exposure through health literacy. The path between attitude and health literacy (*β* = 0.10, *p* = 0.350) was statistically insignificant (Table 3).

### Relationships between knowledge, attitude, and behaviour through nutritional literacy (third model)

The third model indicates the relationship between knowledge, attitude, and behavior and the effect of nutritional literacy on this relationship. For the indirect effect of knowledge, attitude, and behavior through nutritional literacy the model fit values were at acceptable level (CMIN/df = 1.011, NFI = 0.743, CFI = 0.996 and RMSEA = 0.006). The results showed a significant direct path between knowledge, nutritional literacy (*β* = 0.21, *P* = 0.020), and positive effect of nutritional literacy on sunlight exposure (*β* = 0.16, *P* = 0.009). This result revealed that the relationship between knowledge and sunlight exposure might be nutritional literacy dependent. The path between attitudes, and nutritional literacy (*β* = 0.05, *p* = 0.409) was not statistically significant (Table 3).

### Relationships between knowledge, attitude, and sun exposure behaviour through health literacy and nutritional literacy (final model)

**Fig. 1:**
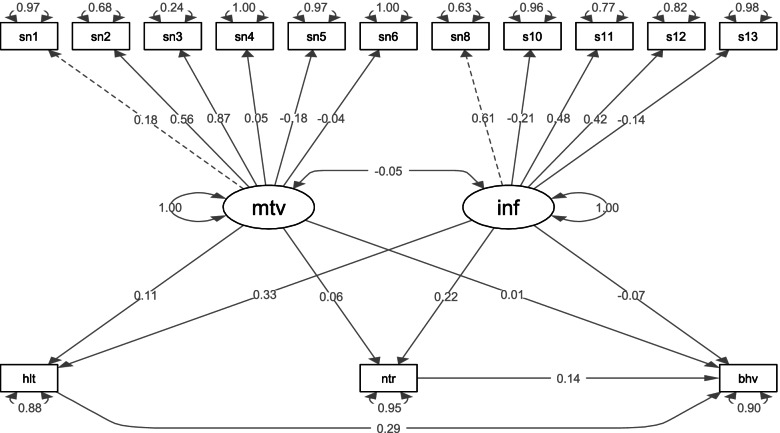
Testing whether health and nutritional literacy is a mediator of the relationships between knowledge and sun exposure and between attitude and sun exposure. Sun1: I like sunlight. Sun2: I use sunhat when exposed to sunlight. Sun3: I use sunscreen products containing SPF ≥ 15 when exposed to sunlight. Sun4: I like outdoor activities. Sun5: Usually I spend most of my time outdoors. Sun6: The time I expose myself to sunlight is enough. Sun7: I have ever heard about vitamin D. Sun8: Vitamin D is good for bone health. Sun9: Vitamin D supports calcium absorption. Sun10: Vitamin D can be supplemented by sunlight exposure. Sun11: The minimum time needed for sunlight exposure is 30 min if we want our body to develop a sufficient amount of vitamin D

The final model shows the relationship between knowledge, attitude, and sun exposure behavior and the effect of health and nutritional literacy on this relationship (Fig. 1). Results of the analysis indicated that there was a relationship between knowledge and sun exposure behavior and health literacy (knowledge and health literacy: *β* = 0.33, *p* < 0.001 and health literacy and sun exposure: *β* = 0.29, *p* < 0.001) and knowledge and sun exposure behavior and nutritional literacy (knowledge and nutritional literacy: *β* = 0.22, *p* = 0.013 and nutritional literacy and sun exposure: *β* = 0.14, *p* = 0.027). Indeed, these results indicate that the relationship between knowledge and sun exposure might be health literacy and nutritional literacy dependent. There was not a significant relationship between attitude and sun exposure even engaging health literacy (attitude and health literacy: *β* = 0.11, *p* = 0.165 and health literacy and sun exposure: *β* = 0.29, *p* < 0.001) and nutritional literacy (attitude and nutritional literacy: *β* = 0.06, *p* = 0.429 and nutritional literacy and sun exposure: *β* = 0.14, *p* = 0.027).

Examination of the path coefficients in the final model showed that there is no direct relationship between knowledge and sun exposure behavior (*β* = -0.07, *P* = 0.47) and attitude and sun exposure behavior (*β* = 0.01, *P* = 0.91). The results of the present study also showed that some paths such as using sun hat and attitudes (*β* = 0.56, *p* < 0.001) and using sunscreen and attitudes (*β* = 0.87, *p* < 0.001) were statistically significant. There was not significant relationship between enjoy outdoor activities (*β* = 0.051, *p* = 0.5), spending time outdoor (*β* = -0.18, *p* = 0.06) and feel have sufficient sun exposure (*β* =- 0.04, *p* = 0.56) and attitudes. Likewise, the relationship between knowledge and three items: bone health (*β* = -0.21, *p* < 0.001), calcium absorption (*β* = 0.01, *p* = 0.48) and supplement vitamin D from sun exposure (*β* = 0.42, *p* < 0.001) were significant, whereas the path of between the minimum time for sun exposure and knowledge (*β* = -0.14, *P* = 0.1) was not statistically significant. The final model showed adequate goodness of fit for the data (RMSEA = 0.040, CIF = 0.85, NFI = 0.657, CMIN/df, = 1.422).

## Discussion

To the best of our knowledge, the current cross-sectional study was the first study to investigate the relationship between health literacy, nutritional literacy, vitamin D knowledge, motivation toward sun exposure, and sun exposure behavior among adults. Since sun exposure is influenced by various factors we thought the structural equation modelling (SEM) would be appropriate solution to achieve the study objective. The SEM allowed this study to examine the complexity of the relationship between health literacy, and nutritional literacy and influence vitamin D knowledge, motivation toward sunlight and sun exposure behavior.

The findings showed that knowledge of vitamin D was associated with sun exposure via health literacy and nutritional literacy. In addition, we found that health literacy and nutritional literacy were positively associated with sun exposure behaviour. In this context, improving health literacy and nutritional literacy might be a useful way to increase the sun exposure behaviour in adults. Despite the lack of enough vitamin D intake [38], there is evidence that people already have good knowledge about vitamin D, beliefs and attitudes towards sunlight [39]. In our study, almost all participants heard about vitamin D, which was in line with a study by Kung and Lee [32] conducted in Chinese women. In contrast to our findings, studies in England [40] and Saudi Arabia [18], and Canada [33] have reported that people had poor knowledge. The results of previous studies on health behaviours showed that health literacy and nutritional literacy are important factors that lead to behavioural change [41]. In addition, to a direct relationship between health behaviours, health literacy and nutritional literacy play an indirect relationship between knowledge and behaviour [19]. A study with a large sample size in China showed that there was relationships between health literacy, knowledge, motivation, and behaviour [42]. Also, in some studies, no direct relationship between knowledge and sun exposure was observed. For instance, a study showed that even among university students with proper knowledge, the use of photo-protective measures was very low [43]. In contrast to our findings, others found a direct relationship between the knowledge of vitamin D and exposure to sunlight [44].

The results also revealed no significant association between motivation and sun exposure. Additionally, health and nutritional literacy did not influence the relationship between motivation and sun exposure. The results showed that individuals’ motivation was not as good as their knowledge of vitamin D and was moderately low. The study showed that less than half of the individuals had a positive attitude toward sun exposure. Similar to our study, others from China [32] and Vietnam [45] have reported a negative attitude toward sunlight. In contrast the results of some studies indicated that people had a positive attitude toward exposure to sunlight [18, 39]. The vitamin D status also is very different in European, Asian, and Middle Eastern countries [46]. However, comparisons with other countries are difficult due to cultural differences in sun exposure. This difference might be attributed to various reasons, including diet, air pollution, and limited sun exposure. We did not investigate the reasons for sunlight avoidance in this study, but the explanation for the negative attitude could be due to cultural factors and the fact that our sample used to “cover-up” tradition (common among Muslim women) that prevents skin contact with UV-B radiation, which is essential for vitamin D production [47]. Another reason might be attributed to knowing the harmful effects of sunlight (e.g., aging skin, darkening of the skin, and skin cancer) [48]. On the other hand, the use of sunscreens, sunglasses, or sun hat is a factor in reducing sun exposure among adults [49]. Also, genetic variation could cause differences in vitamin D levels among people. The vitamin D receptor (VDR) gene plays an important role in vitamin D metabolism. Polymorphisms in this gene can affect vitamin D expression, control vitamin D metabolism by hydroxylase enzymes, or cause problems in the vitamin D binding protein (DBP) [50]. In addition, skin type genetically affects the amount of vitamin D3 that can be synthesized in the skin for a given dose of sun exposure [51]. Moreover, studies showed that an inverse association between obesity and serum vitamin D3 exists. Indeed, adipose tissue may trap circulating 25OHD concentration leading to vitamin D deficiency [52]. Also, the capacity of the skin to produce vitamin D with age decreases [53].

This study had several strengths. The current study was the first to examine the relationship between health literacy, nutritional literacy, knowledge, motivation, and sun exposure among adults using SEM. Also, the study included a large sample size with a variety of ages, professions, and educational backgrounds. However, we should also consider a few limitations. First, causality cannot be inferred by the cross-sectional nature of this study. Longitudinal and experimental approaches are needed to further explore the relationship between health literacy, nutritional literacy, knowledge, motivation, and sun exposure. Second, the use of vitamin D supplements should be further investigated because vitamin D supplements may be a negative motivation for sun exposure. Third, since this study has been conducted in Iran, whose lifestyle and cultural context may be different from those of other countries, the current findings may differ from those that do not match the rest of the world. Finally, we did not look at the reasons for sun exposure avoidance.

## Conclusions

The findings of the current study showed that health literacy and nutritional literacy were significantly associated with knowledge but not significantly associated with attitudes toward sunlight exposure. Rather, health literacy and nutritional literacy mediated the relationships between knowledge and sunlight exposure. The findings also suggest that health professionals should consider the level of health literacy and nutritional literacy of individuals when conducting health education on sun exposure as a way to receive vitamin D.

## Data Availability

The data supporting the findings of this study are available from the corresponding author on request.

## Abbreviations

SEM: Structural Equation Modelling
BMI: Body mass index
HELIA: Health Literacy Instrument for Adults
NLS: Nutrition Literacy Scale
NFI: Normed fit index
CFI: Comparative fit index
RMSEA: Root-mean-squared error associated
IPAQ: International physical activity questionnaire
SPF: Sun Protection Factor
BP: Blood pressure
VDR: Vitamin D receptor
DBP: Vitamin D binding protein
